# Ion channels or aquaporins as novel molecular targets in gastric cancer

**DOI:** 10.1186/s12943-017-0622-y

**Published:** 2017-03-06

**Authors:** Jianling Xia, Hongqiang Wang, Shi Li, Qinghui Wu, Li Sun, Hongxiang Huang, Ming Zeng

**Affiliations:** 1 Cancer Center, Sichuan Academy of Medical Sciences and Sichuan Provincial People’s Hospital, Hospital of the University of Electronic Science and Technology of China, The Western First Round Road, Section 2#32, Chengdu, 610072 China; 2 Department of Oncology, Zhoushan Hospital, Zhoushan, 316000 China; 3 Department of Urology, The First Affiliated Hospital of Wenzhou Medical University, Wenzhou, 325015 China; 4 Department of Urology, Hainan Provincial People’s Hospital, Haikou, 570311 China; 5 Department of Oncology, Nanfang Hospital, Southern Medical University, Guangzhou, 510515 China

**Keywords:** Ion channels, Potassium, Chloride, Calcium, Sodium, Aquaporin, Gastric cancer, Therapeutic target

## Abstract

Gastric cancer (GC) is a common disease with few effective treatment choices and poor prognosis, and has the second-highest mortality rates among all cancers worldwide. Dysregulation and/or malfunction of ion channels or aquaporins (AQPs) are common in various human cancers. Furthermore, ion channels are involved in numerous important aspects of the tumor aggressive phonotype, such as proliferation, cell cycle, apoptosis, motility, migration, and invasion. Indeed, by localizing in the plasma membrane, ion channels or AQPs can sense and respond to extracellular environment changes; thus, they play a crucial role in cell signaling and cancer progression. These findings have expanded a new area of pharmaceutical exploration for various types of cancer, including GC. The involvement of multiple ion channels, such as voltage-gated potassium and sodium channels, intracellular chloride channels, ‘transient receptor potential’ channels, and AQPs, which have been shown to facilitate the pathogenesis of other tumors, also plays a role in GC. In this review, an overview of ion channel and aquaporin expression and function in carcinogenesis of GC is presented. Studies of ion channels or AQPs will advance our understanding of the molecular genesis of GC and may identify novel and effective targets for the clinical application of GC.

## Background

Gastric cancer (GC) remains as a highly lethal malignancy worldwide, particular for developing country, which makes it a key public health problem [1–3]. High incidence regions include Asia, Eastern Europe and Middle and South America [4]. Complete remission of early GC by surgical or even minimal invasion endoscopic resection removal is now possible as a result of developments in therapeutic techniques [5]. Nevertheless, the majority of GC patients are asymptomatic only until entering an advanced stage. Common therapeutic options include surgery, radiotherapy, and chemotherapy [6, 7]. Studies have shown that patients with advanced stage GC have a poor prognosis with a 5-year survival rate less than 30% [8]. Recently, advances in the biology and molecular profiling of GC have resulted in targeted treatments and better survival rates in select patients with advanced GC. Trastuzumab and ramucirumab are two new therapeutic targeted drugs that have been approved in the last 5 years for the treatment of advanced GC [9]. However, the development of more highly effective and selective targeted agents is still an important issue for the appropriate management of advanced GC and more research is needed to further improve outcome.

**Fig. 1:**
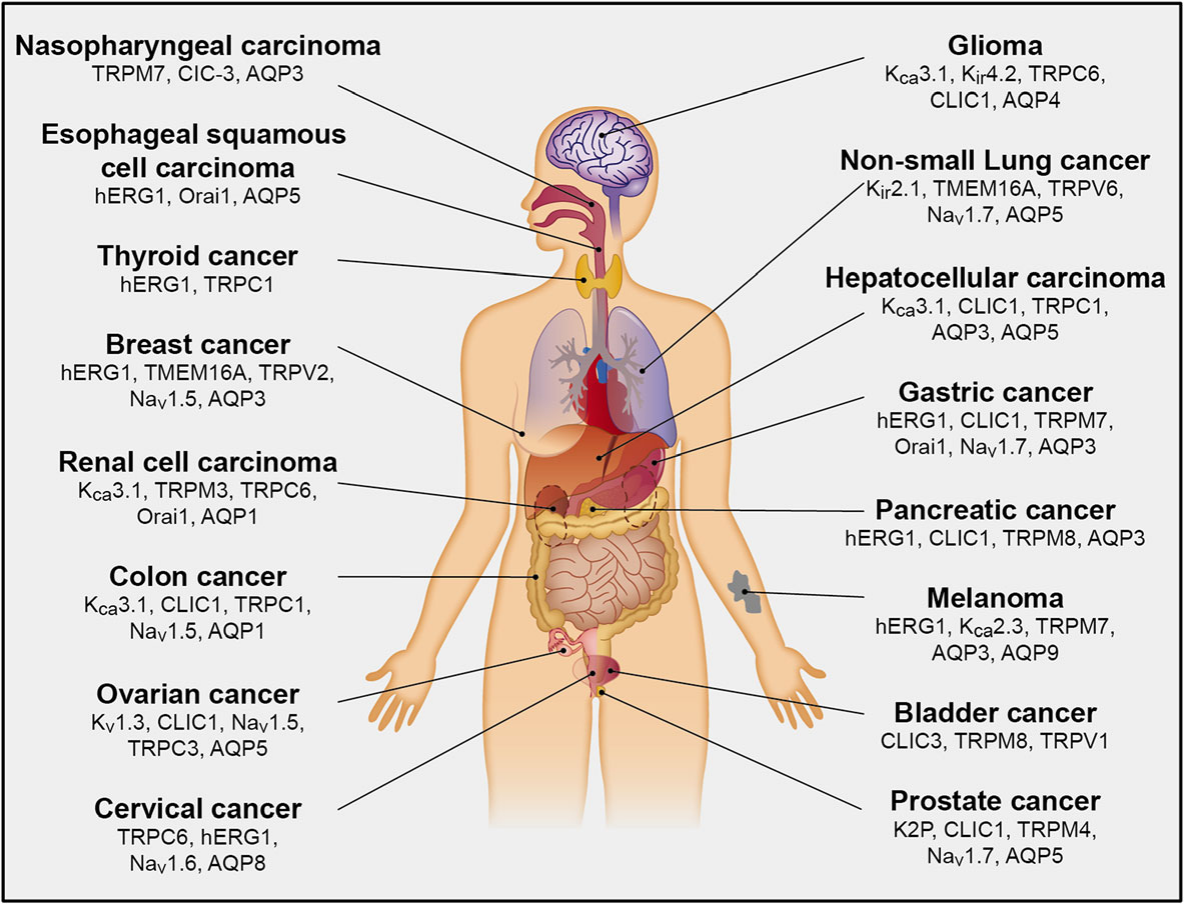
Representative ion channels or aquaporins associated with malignant behavior of tumor cells

**Fig. 2:**
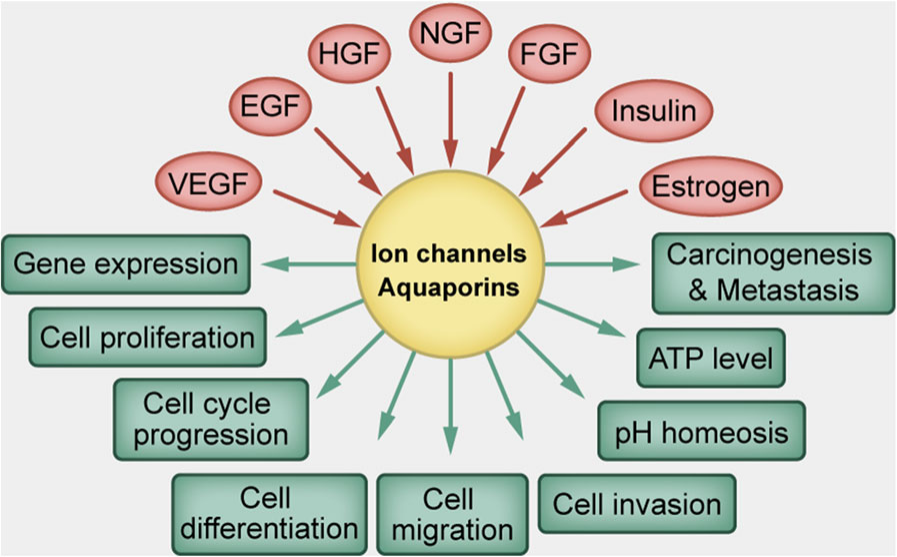
Multiple characteristics of ion channels and aquaporins under different stimuli and showing different responses

Ion channels and aquaporins (AQPs) are comprised of transmembrane proteins that regulate the permeation of specific ions or water between the extracellular and intracellular environments. Ion channels or AQPs form rapid cell signals in excitatory tissues by modifying ion fluxes at various sites within cells on various time scales. Ion channels and AQPs also contributed to slower processes, such as volume regulation, proliferation, apoptosis, migration, invasion and cell adhesion in non-excitable cells [10–14]. It has become increasingly clear over the last 15 years that splicing, dysregulated expression and/or function of ion channels and/or AQPs exists in many types of cancers and this leads to multiple cancer processes (Figs. 1 and 2). In addition, ion channels and AQPs may modulate cell activities and control many key aspects of neoplastic progression. They may also determine the fate of these cells. Therefore, ion channels that have an impact on cancer development and/or progression are called “oncogenic channels” [15]. Many studies from different laboratories have shown that adjustment of ion channels or AQPs by either altered expression or inhibited activity can weaken the growth and/or migration of cancer cells. This means ion channels or AQPs could become pharmaceutic targets in GC management [16].

The primary aim of this review was to provide an update of recent advances on the role of ion channels and AQPs in GC. The final objective was to assess the potential use of ion channels in the clinical management of GC and to evaluate the possibility of ion channels to be pharmacological targets for GC.

## Potassium channels

Potassium (K^+^) channels have the greatest amount of diversity among ion channels and aquaporin in the plasma membrane, which are encoded by over 75 distinct genes. There are four main classes according to their activation mechanisms and domain structure. They are: calcium-activated K^+^ channels (K_Ca_), voltage-gated K^+^ channels (K_v_), inward-rectifier K^+^ channels (K_ir_) and two-pore-domain K^+^ channels (K2P). The voltage-dependent K^+^ channel (K_v_) family can be subdivided into K_v_1-4 channels (Shaker, Shab, Shaw and Shal-like subunits), the silent K_v_5, K_v_6, K_v_8 and K_v_9 subunits (regulators), K_v_7 channels (KCNQ), and K_v_10-12 channels (EAG-like) [17, 18]. Potassium channels in non-excitable cells are likely involved in mediating intracellular Ca^2+^ concentrations, cell volume, pH homeosis, apoptosis, cell cycle and differentiation [19]. Many studies have shown that ectopic expression of K^+^ channels occurs in human cancers. There are four main classes of K^+^ channels that have been involved in oncological processes [20]. As an example, intermediate-conductance calcium-activated potassium (SK4) channels have been identified to overexpress in triple-negative breast cancer. They are also implicated in proliferation, apoptosis, migration and epithelial-to-mesenchymal transition (EMT) processes of cancer cells [21]. K_ir_2.1 regulates tumor cell proliferation and drug resistance by modulation of multidrug resistance protein 1 (MRP1). It is also simultaneously regulated by miR-7 and the Ras/mitogen-activated protein kinase (MAPK) pathway in small-cell lung cancer [22]. Meanwhile, it has been shown that TREK-1, which is in the K2P family, is overexpressed in prostate cancer. High levels of TREK-1 were positively related to T staging and Gleason scores. They were also associated with shorter castration resistance free survival. In addition, TREK-1 knockdown significantly attenuated prostate cancer cell proliferation both in vitro and in vivo. Also, TREK-1 induced a G1/S cell cycle arrest [23]. K_v_9.3 in colon cancer showed similar results [24].

Various K^+^ channels have been shown to play an important role in GC progression, particularly the human enter a go-go related potassium channel (hERG1), which is also known as K_v_11.1 and belongs to the EAG family. hERG1 has been demonstrated to be negatively expressed in the surrounding non-cancerous tissues; however, this switched to abnormally positive expression in GC tissues according to immunohistochemistry analysis. This indicated that hERG1 is a potential biomarker for GC [25]. hERG1 expression levels have also been correlated with TNM stage, grading, lymph node involvement, tumor differentiation and vascular endothelial growth factor (VEGF)-A expression. hERG1 has also been shown to enhance GC cell invasion and proliferation, induce cell cycle progression in vitro, and promote tumor genesis and growth in vivo. Furthermore, hERG1 modulates VEGF-1 secretion through an AKT-dependent pathway [26, 27]. Of significance, hERG1 has been demonstrated to be necessary for cisplatin induction of apoptosis in GC and may provide a new potential target for cisplatin chemotherapy [28]. In addition to hERG1 participating in GC cell behavior, other K^+^ channels are also crucial in GC. Kir2.2 knockdown results in senescence-induced mitochondrial dysfunction. This dysfunction includes increases in the mitochondrial mass and reactive oxygen species (ROS) production, as well as decreases in the mitochondrial membrane potential and ATP levels in GC [29]. Also, K_v_4.1 was demonstrated to affect cell cycle distribution and facilitate cell proliferation [30]. K_Ca_ (the detailed subtype is not known) is possibly involved in hepatocyte growth factor (HGF)-induced GC cell proliferation [31]. Similarly, KCNQ1 and K_v_1.5 were identified to be involved in GC progression [32, 33]. There have not been any reports, however, on the role of K2P in GC.

In summary, different K^+^ channels can remarkably affect various aspects of GC cell behaviors both in vitro and in vivo. Therefore, the clinical potential of K^+^ channels should be studied further, especially regarding underlying cell signaling pathways.

## Chloride channels

In a study by Jentsch et al., chloride (Cl^−^) channels were divided into 6 categories based on the opening mechanisms of the channels: 1) the cystic fibrosis transmembrane conductance regulator (CFTR), which is a cAMP-activated Cl^−^ channel, 2) voltage-gated Cl^−^ channels (ClCs), which are gated in a voltage-reliant manner, 3) Ca^2+^-activated Cl^−^ channels (CACCs), which are dependent on intracellular calcium, 4) volume-regulated Cl^−^ channels (VRACs), which are also called swelling-activated Cl^−^ channels, 5) a family of p64-related proteins (CLICs), which are putative intracellular Cl^−^ channels, and (6) γ-aminobutyric acid and glycine receptors, which are ligand-gated Cl^−^ channels. The primary functions of Cl^−^ channels are regulation of trans-epithelial transport, volume (osmoregulation), membrane potentials and cellular immune responses [34]. They were shown to be critical for tumor progression in several studies. C1C3 has been proposed to promote cervical cancer cell membrane ruffling, which is required for tumor metastasis and may be a valuable prognostic biomarker and therapeutic target [35]. Studies on nasopharyngeal carcinoma cells showed similar results [36]. Meanwhile, Ca^2+^-activated Cl^−^ channel transmembrane protein 16A (TMEM16A, also known as ANO1) could promote breast cancer progression by activating calmodulin-dependent protein kinase (CAMK) and epidermal growth factor receptor (EGFR) signaling [37]. It may also act on cell invasion and proliferation, and tumor growth in non-small cell lung cancer [38]. In colon and pancreatic cancer, it seems that CLIC1 may be a putative oncogene and may play a pivotal role in tumor progression [39, 40].

In GC, intracellular Cl^−^ has been suggested to regulate lysosomal acidification and autophagy functions, which indicated an important role of Cl^−^ in GC progression [41]. CLIC1 has been shown to be involved in GC development and, importantly, CLIC1 was considered to be a possible prognostic marker for GC. Its expression levels in GC samples were increased relative to adjacent noncancerous mucosa, and were associated with the TNM stage, perineural invasion, lymph node metastasis and a 5-year survival rate [42]. In addition, elevated CLIC1 expression was implicated to accelerate the invasion and migration of GC cells in vitro. This is possibly mediated by proteasome activator 28β subunit (PA28β), which then affects the ROS-mediated p38 MAPK signaling pathway [43, 44]. However, it is intriguing that high CLIC1 expression could also enhance apoptosis and inhibit proliferation [45]. There is also evidence that TMEM16A was involved in GC progression. TMEM16A was significantly amplified and upregulated in GC specimens. The overexpression of TMEM16A was positively associated with disease stage and negatively associated with patient survival, identifying it as an independent prognostic factor for patient outcome [46]. The upregulation of TMEM16A could be due to the transcriptional factor and activator of transcription 6 (STAT6) binding to its promoter region in response to IL-4 [47]. Few studies, however, have investigated how TMEM16A modulated downstream activity.

In conclusion, the involvement of CLIC1 and TMEM16A channels in GC cell migration, invasion and disease progression has been demonstrated. Nevertheless, more studies are required to determine whether other Cl^−^ channels play a role in GC progression. Also, studies are needed to increase knowledge of the mechanistic side of Cl^−^ channel pathophysiology and in vivo experiments should be performed.

## Calcium channels

The “transient receptor potential” (TRP) channels include a wide variety of ion channels. The majority are permeable to divalent and monovalent cations and they influence calcium (Ca^2+^) homeostasis. There are seven subfamilies of TRP channels that comprise the vanilloid receptor related (TRPV), classical (TRPC), and melastatin-related (TRPM) channels [48, 49]. These channels play an important role in the cell cycle, which often occurs by regulating gene transcription. Also, these channels influence other cellular processes such as apoptosis, proliferation and cell motility [50]. An increasing number of reports have shown TRP channels are involved in tumor progression. In breast cancer, high levels of TRPM7 have been confirmed to independently predict poor outcomes and TRPM7 is functionally required for metastasis in a mouse xenograft model. In terms of mechanisms, TRPM7 regulates myosin II-based cellular tension. This then regulates the focal adhesion number, polarized cell movement and cell-cell adhesion [51]. Meanwhile, TRPV2 was higher in prostate cancer with metastatic cancer (stage M1) relative to primary solid tumors (stages T2a and T2b). TRPV2 has been demonstrated to facilitate the growth of prostate cancer cells and their invasive properties. It also upregulated expression of invasive enzymes such as matrix metalloproteinase-2 (MMP2), MMP9 and cathepsin B in vitro and in nude mice [52]. Furthermore, TRPC6 was shown to be essential for glioma development through modulation of G2/M phase transition. It therefore could be a new target for glioma therapeutic intervention [53].

Store operated Ca^2+^ entry (SOCE), which is constituted by pore forming Ca^2+^ channel subunits Orai1, Orai2 and/or Orai3, and also their modulators STIM1 and/or STIM2, is critical for Ca^2+^ oscillation, except for the TRP channels that have been implicated in Ca^2+^ homeostasis in cells [54]. Orai1 and STIM1 have been shown to be highly expressed in tumor cells, and may contribute to malignant biological behaviors. For example, Orai1/STIM1 expression and/or function are elevated in therapy resistant ovary carcinoma cells, which is at least in part due to increased AKT activity. Also, this may influence therapy resistance in these cells [55]. In addition, Orai1 and STIM1 are crucial for breast cancer cell migration in vitro and tumor metastasis in vivo [56].

In GC, TRPM7 plays an influential role in the growth and survival of GC cells. It can also depress cell apoptosis and is likely to be a potential target for pharmacological therapy of GC [57, 58]. TRPV6 mediates capsaicin-induced apoptosis in GC cells. The abundance of TRPV6 in GC cells determines life or death after capsaicin treatment [59]. The TRPC6 channels are essential for GC cell proliferation and the G2/M phase transition in vitro, and also for the development of GC in nude mice [60]. Moreover, we showed that intracellular Ca^2+^ is elevated in GC cells. Also, store-operated Ca^2+^ currents are found in GC cells. The composite molecules of SOCE; i.e., Orai1 and STIM1 suggest a poor outcome for GC by advancing tumor cell proliferation, metabolism, migration, and invasion through targeting metastasis-associated in colon cancer-1 (MACC1) [61].

In summary, the data suggest that upregulation of certain subtypes of Ca^2+^ channels contribute to GC tumorigenesis, progression and, possibly, drug resistance. Ca^2+^ channels are therefore potential drug targets for GC.

## Voltage-gated sodium channels

Voltage-gated sodium (Na^+^) channels (VGSCs) consist of a pore-forming α subunit, typically crosslinking with one or more, identical or different, smaller β subunits [62]. Nine genes in humans (*SCN1A* to *SCN5A*, and *SCN8A* to *SCN11A*) code for nine distinct VGSC proteins (Na_v_1.1 to Na_v_1.9, respectively) associated with differences in their α subunits [63]. These proteins allow voltage-dependent activation of sodium current, and are also responsible for membrane depolarization, which is thought to be specific in cells characterized as being “excitable”, such as skeletal cells, cardiac muscle cells and neurons [64]. Over the last 15 years, an increasing number of studies have shown the expression of these channels in non-excitable cells. In these cells, they affect physiological functions such as endocytosis, phagocytosis, secretion, motility, and cell proliferation and differentiation [65–68]. There has been a rapid expansion of published studies documenting the expression of VGSCs in many cancers. Also, their role in the regulation of cellular invasion and migration and, importantly, their potential use as diagnostic and/or therapeutic targets has also been studied [69]. In colon cancer, Na_v_1.5 is overexpressed and is a vital controller of a gene transcriptional system that regulates cell invasion [70]. In non-small cell lung cancer, Na_v_17 is required for the epidermal growth factor (EGF)-mediated extracellular signal-regulated kinase 1/2 (ERK1/2) pathway to enhance cell invasion [71]. Likewise, in cervical cancer, overexpressed Na_v_1.6 has been correlated with increased tumor cell invasion [72].

In GC, our results indicated that Na_v_1.7 was the most abundantly expressed VGSC subtype in both GC tissues and GC cell lines. Na_v_1.7 expression was shown to be frequently higher in GC tissues compared to non-malignant samples. Na_v_1.7 expression was correlated to GC patient prognosis and also with the transporter Na^+^/H^+^ exchanger-1 (NHE1) and the oncoprotein MACC1 expression. Inhibition of Na_v_1.7 led to reduced NHE1 expression. This ultimately resulted in a slower rate of GC cells invasion and proliferation in vitro and tumor growth in nude mice. Na_v_1.7 suppression was also associated with decreased expression of MACC1, and MACC1 suppression resulted in decreased NHE1 expression. The study results demonstrated that Na_v_1.7 controls GC cell invasion and proliferation by MACC1-mediated upregulation of NHE1. Therapies that specifically target Na_v_1.7 might successfully impede GC progression [73]. However, there have been no other reports on VGSCs in GC.

In summary, despite that our data suggested that functional Na_v_1.7 expression have broad influence on the pathophysiology of GC, the available evidence remains limited. Further studies on both the basic and clinical aspects are needed. Also, these studies should extend to other intracellular mechanisms and assess whether Na_v_1.7 is related to drug resistance.

## AQPs

AQPs are part of a special superfamily of membrane integral proteins, which are known as major intrinsic proteins. AQPs can transport water and sometimes water and glycerol (“aquaglyceroporins”). They therefore can regulate cell volumes and can regulate body water homeostasis [74, 75]. AQP0 to AQP12 of this family were reported. They were divided into three subgroups based on their main sequences: water selective (AQP0, 1, 2, 4, 5, 6, and 8), aquaglyceroporins (AQP3, 7, 9, and 10), and superaquaporins (AQP11 and 12) [76]. AQPs have been shown to be crucial for malignancy. AQP3, for example, induced ERK1/2 activation. This then increases MMP-3 expression and secretion, and therefore controls prostate cancer cell invasion and motility [77]. AQP4 has been implicated to be upregulated in glioma specimens and plays a critical role in glioma-associated edema [78]. AQP5 has been shown to be overexpressed in breast cancer and it possibly acts on cell proliferation and migration [79]. In addition, AQP8 has been shown to be involved in cervical cancer progression [80].

GC tissues express obviously higher levels of AQP3 compared to normal gastric mucosa. Also, upregulation of AQP3 was related to EMT-associated proteins and may predict poor outcome for GC. AQP3 regulated GC cell proliferation, invasion and migration. It also can induce an alteration in expression levels of EMT-related proteins and MMPs through the PI3K/AKT/SNAIL signaling pathway in vitro [81]. In addition, AQP3 can transport glycerol, which is required for GC cell energy production and lipid synthesis [82]. As for its upstream, a study reported that c-Met could modulate AQP3 expression through the ERK1/2 signaling pathway in GC [83]. Moreover, miR-874 suppresses AQP3 expression by binding to the 3^’^UTR of AQP3 mRNA in GC cells [84]. Also, human EGF induced AQP3 expression in a time- and dose-dependent manner in GC cells [85]. An additional AQP subtype that has been demonstrated to play a role in malignant biological behaviors of GC cells is AQP5. The overexpression of AQP5 was observed in GC tissues relative to the paired normal tissues, according to a study by Huang et al. This upregulation was correlated with enhanced lymph node metastasis according to immunohistochemistry. In vitro, overexpression of AQP5 significantly promoted, while knockdown of AQP5 notably inhibited the capacity of GC cell proliferation and migration [86]. Also, AQP5 upregulation may play a role in GC cell differentiation [87]. These results indicated that modulation of AQP5 expression or function may be a potential treatment for GC even though the detailed mechanisms by which AQP5 promotes GC progression is unknown.

In conclusion, there is a possibility that AQP channels have clinical potential for the treatment of GC, as is the case with other cancers. Yet, further studies are needed to reveal other subtypes of AQP channels that participate in tumor progression and provide insight into the pathophysiological aspects of GC more deeply. Also, more in vivo experiments are required to support the above-mentioned findings.

## Pharmacological targeting of ion channels and AQPs for cancer therapy

The above paragraphs have shown the crucial role of ion channels and AQPs in tumor progression. Thus, using specific pharmacological modulators of ion channels or AQPs may pave the way for novel cancer therapy. For example, treatment with margatoxin (MgTX), which is a selective inhibitor of K_v_1.3, or short hairpin RNA against K_v_1.3, notably attenuated non-small cell lung cancer cell proliferation and altered cell cycle progression. The use of MgTX in a lung adenocarcinoma model for 7 days obviously lessened tumor volume by 75%. Injection of MgTX into the tumor tissues of nude mice significantly increased p21 expression level and significantly decreased cycline D3 and CDK4 expression levels, indicating this blocker controls the cell cycle at G1-S in vivo, which was similar to the in vitro cultured system [88]. Likewise, as an antagonist of TRPM8, cannabigerol (CBG) inhibited cell proliferation, induced cell apoptosis and stimulated ROS production in colon cancer cells. In an in vivo study, nude mice were subcutaneously injected with colon cancers cells. CBG (3 mg/kg or 10 mg/kg given intraperitoneally every day) used as treatment produced a dramatic reduction from day 3 of treatment to the end of the experiment [89]. Also, the VGSC-blocking drug phenytoin suppressed the invasion and migration of breast cancer cells in vitro. Phenytoin treatment also significantly reduced cancer cell invasion into the surrounding mammary tissue and tumor growth in vivo. Phenytoin significantly inhibited metastasis to the lungs, liver and spleen [90]. In addition, a decrease in CLIC1-mediated Cl^−^ currents and glioblastoma cell proliferation were also seen with CLIC1 antibody addition. Meanwhile, glioblastoma cells exposed toisotype control antibodies or CLIC1 antibodies were transplanted into the brains of immunodeficient mice. CLIC1 antibody treatment led to smaller tumors and significantly improved overall survival in mice. This indicated treatment with CLIC1 antibodies produces an obvious decrease in in vivo tumorigenicity of glioblastoma cells [91].

**Fig. 3:**
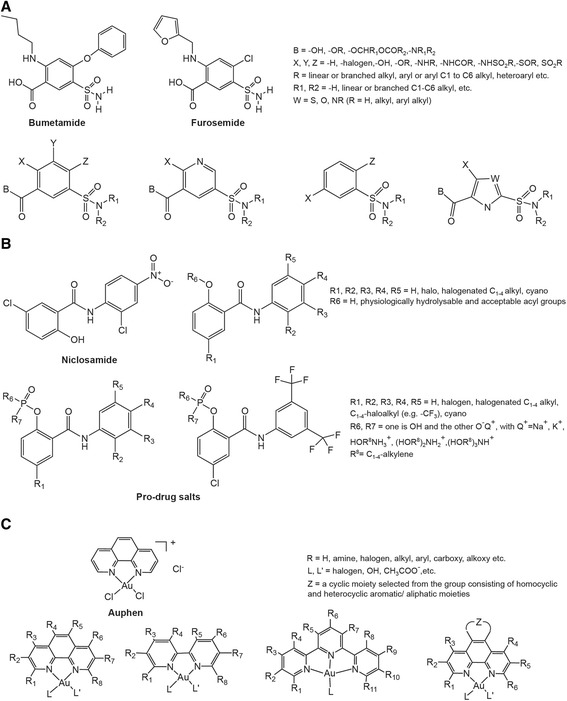
The structures of some aquaporin inhibitors are shown. **a** Structure of sulfonamide-based compounds that act as AQP1 and AP4 modulators. **b** Structure of AQP2 and AQP4 phenylbenzamide-type inhibitors and associated pro-drug salts. **c** Gold(III) complexes that can be selective aquaglyceroporin inhibitors

There are a number of inhibitors of AQPs that may be useful in treating tumors other than the above-mentioned inhibitors of ion channels (the structure of some inhibitors of AQPs are shown in Fig. 3) [92–94]. In colon cancer, for example, inhibition of AQP1 by AqB013 effectively reduced tumor cell migration and invasion as well as endothelial tube formation [95]. In pancreatic cancer, inhibition of AQP3 by the inhibitor CuSO4 attenuated tumor cell migration, which suggested it was a potential therapeutic treatment agent for pancreatic cancer [96]. Meanwhile, there are a few patents that have been filed that represent small molecule AQP modulators and pharmaceutical formulations, such as WO2013005170 (metal-based inhibitor of aquaglyceroporins AQP3, AQP7, and AQP9), US8835491 (modulator of orthodox aquaporin AQP1), and WO2008052190 (modulators of orthodox aquaporin AQP4) that may be used in therapeutic applications in the clinic [92].

As illustrated in the above examples of other types of cancer, this relatively new area of study has already led to the identification of potential therapeutic drugs for GC. With the use of hERG1 inhibitor E4031 daily for 2 weeks starting from the day after inoculum in in vivo experiments, the masses were notably decreased with E4031 treatment, which was paralleled by the decrease in tumor angiogenesis. In addition, within the masses in E4031-treated mice, there was a reduction in VEGF-A expression and AKT prosphoralation, which strongly confirmed the in vitro data [26]. SKF96365, an agent known to block TRPC channels, also stagnated the GC cell cycle in the G2/M phase and inhibited cell proliferation in vitro. Application of SKF96365 (20 mg/kg) for 5 successive days through intraperitoneal injection after 7-day implantation led to reduced tumor volume in vivo [60]. Our study showed the VGSC inhibitor tetrodotoxin (TTX) decreased MACC1 and NHE1 expressions, which decreased the extracellular pH and increased the intracellular pH. This affected the GC cell proliferation and invasion in vitro [73]. However, we did not use TTX in the experiments on mice, which should be done in the future.

Since the set of ion channels that promote GC development is now known, it is imperative to conduct in-depth research on the specific ion-channel targeting drugs. Furthermore, the potential side-effects should be addressed.

## Conclusions and future perspectives

### Conclusions

The primary conclusions of this review are the following: 1) many ion channels and AQPs are differentially expressed in GC tissues and cells relative to normal gastric mucosal specimens and cells. 2) Changes in the expression or activity of ion channels or AQPs give the GC cells a pathological character and the ion channels then affect various aspects of the malignant behavior of GC cells, such as proliferation, migration and invasion. 3) Multiple signaling pathways may be activated by ion channels and AQPs and this could enhance a variety of oncogenes, thereby contributing to GC tumorigenesis and progression. 4) Inhibiting the expression or blocking the activity of ion channels or AQPs impairs GC cell function both in vitro and in vivo, which could open a new avenue for pharmaceutical research in GC. We therefore suggest that ion channels could be realistic, functional biomarkers and new therapeutic targets in GC, as in many other cancers.

### Future perspectives

Much more work needs to be done on GC from multiple aspects. Firstly, the ion channels or AQPs that have already received attention require further characterization. Moreover, other ion channels or AQPs that might be possibly important are worth investigation. Secondly, angiogenesis, lymphangiogenesis, autophagy dysregulation, and the Warburg effect are ubiquitous in GC, and these phenomena are regulated by ion channels and/or AQPs in many other types of cancer. Therefore, whether ion channels and/or AQPs affect the above-mentioned phenomena in GC should also be determined. Crociani et al. showed that HERG1 regulated VEGF-A expression and angiogenesis in GC and a combined treatment with hERG1 inhibitors and antiangiogenic drugs could enhance the therapeutic effect and repress tumor growth [26]. Dong et al. demonstrated that AQP3 promotes cisplatin resistance in GC cells through autophagy [97]. There have been no additional reports, however, about the relationship between ion channels/AQPs and the other above-mentioned phenomena in the progression of GC. Thirdly, the detailed mechanisms or other factors that are involved in GC processes should be explored further even though some signaling pathways or oncogenes have been confirmed to participate in the contribution of ion channels to GC progression. In addition, ion channels or AQPs may be regulated by growth factors such as EGF, HGF, VEGF, nerve growth factor (NGF) and fibroblast growth factor (FGF), as well as the hormones estrogen and insulin [30, 71, 98–102] (Fig. 2). However, the research on the upstream of the ion channels or AQPs is still limited, which attenuates our understanding of how the ion channels or AQPs are upregulated and/or activated. Fourth, albeit many previous reports showed circumstantial evidence, there is a conspicuous insufficiency of studies that have utilized site-directed mutagenesis to change the biophysical properties of the related currents. Meanwhile, KO mouse models may also assist with establishing the facility of these interesting proteins, which is absent in the present studies. Finally, and importantly, specific blockers or antagonists that specifically act on certain subtypes of ion channels or AQPs and have few side effects should be developed to translate basic research findings into clinical applications. The currently available agents primarily influence a variety of ion channel or aquaporin subtypes; however, these agents are not specific, which can lead to clinically adverse effects that limit their usage. Thus, there is an urgent need to develop drugs that target specific subtypes of ion channels or AQPs. Furthermore, large-scale studies should evaluate whether these agents have protective function over the long-term in GC patients.

In summary, the use of ion channels or AQPs as diagnostic, prognostic, or therapeutic targets for GC is possible. K^+^ channels (hERG1, Kir2.2, K_v_4.1, KCNQ1, and K_v_1.5), Cl^−^ channels (CLIC1, and TMEM16A), Ca^2+^ channels (TRPM7, TRPV6, TRPC6, and Orai1), Na^+^ channels (Na_v_1.7), and AQPs (AQP3, and AQP5) may be potential candidate markers for GC. Further research and rigorous evaluation are needed to determine which is better for use in the clinic. Although progress has been made in this area, much work remains to be conducted. Furthermore, the exploration of pharmacological agents for ion channels may enable the development of therapies for GC.

## Abbreviations

AQP: Aquaporin
CBG: Cannabigerol
EGF: Epidermal growth factor
EMT: Epithelial-to-mesenchymal transition
ERK1/2: Extracellular signal-regulated kinase 1/2
FGF: Fibroblast growth factor
GC: Gastric cancer
HGF: Hepatocyte growth factor
MACC1: Metastasis-associated in colon cancer-1
MAPK: Mitogen-activated protein kinase
MgTX: Margatoxin
MMPs: Matrix metalloproteinases
NGF: Nerve growth factor
NHE1: Na^+^/H^+^ exchanger-1
ROS: Reactive oxygen species
SOCE: The store operated Ca^2+^ entry
TRP channels: ‘Transient receptor potential’ channels
TTX: Tetrodotoxin
VEGF: Vascular endothelial growth factor
VGSCs: Voltage-gated sodium channels
